# Neighbourhood tobacco supply and individual maternal smoking during pregnancy: a fixed-effects longitudinal analysis using routine data

**DOI:** 10.1136/tobaccocontrol-2018-054422

**Published:** 2018-11-02

**Authors:** Tom Clemens, Chris Dibben, Jamie Pearce, Niamh K Shortt

**Affiliations:** School of Geosciences, University of Edinburgh, Edinburgh, UK

**Keywords:** environment, priority/special populations, socioeconomic status

## Abstract

**Background:**

Tobacco policy is increasingly focusing on the ‘tobacco endgame’ which commits to eradicating tobacco use (prevalence below 5%) within the next two decades. Strategies for achieving the endgame are likely to include addressing the supply of tobacco products, yet current evidence to support this approach is primarily cross-sectional.

**Methods:**

We use longitudinal smoking information from routine maternity records of all women who gave birth in Scotland between 2000 and 2015. We linked this data to the residential density of retailers selling tobacco products and the neighbourhood prevalence of smoking during pregnancy. In the analysis, individual mothers act as their own controls because we compare changes in their smoking behaviour between pregnancies to changes in exposure to tobacco retailing that arises from residential movement between pregnancies.

**Results:**

Adjusted ORs showed an increased risk of being a smoker associated with increases in exposure to retailer density (OR 1.67, 95% CI 1.27 to 2.20).

**Conclusions:**

The results provide the strongest evidence to date of an association between the neighbourhood availability of tobacco and smoking, and the first to do so among pregnant women. These findings provide supportive evidence for interventions targeting the supply of tobacco products in achieving the endgame.

## Introduction

Prompted by the slow decline in smoking in developed countries, and the rise in tobacco use in many low-income and middle-income nations, there has been increased international attention aimed at developing effective policy interventions and targets to eradicate the toll of tobacco. A number of policy opportunities have been identified including tax increases for tobacco products, smoking restrictions in non-residential indoor and outdoor locations, and restrictions on the branding and packaging of tobacco products.^1^ An increasingly important focus for intervention has been retail supply and availability.^2^ There is now a significant body of international cross-sectional evidence linking the density of tobacco retailers in residential, school and other settings with smoking outcomes among different sociodemographic groups.^3–7^

Tobacco retailing remains ubiquitous in many countries, particularly in urban areas^8^ and the density of tobacco retailing tends to be higher in more socially deprived communities.^3 8^ Greater geographical availability of tobacco in local communities is important because it is likely to have a role in creating competitive local markets that reduce the price of tobacco products, resulting in higher levels of consumption, and undermining smoking cessation attempts.^9^ Availability of tobacco within walking distance from home has also been shown to be associated with unsuccessful quit attempts indicating how greater availability of tobacco acts to reduce other non-monetary costs of obtaining tobacco.^10^ Further, tobacco retailing enhances the visibility of tobacco products, and provides visual cues to encourage purchasing and smoking. The increased availability and heightened visibility of tobacco products are likely to lead to smoking prevalence being perceived as higher and contribute to the local normalisation of smoking. However, even in jurisdictions such as Scotland, where tobacco products must be hidden from display, signage and shelving units indicating the availability of tobacco remain prominent,^11^ and there is evidence that retailing availability remains important even in these environments where display and promotion is restricted.^12^

While previous international work has been instructive in identifying tobacco retailer density as a possible opportunity for new policy interventions, the work to date has suffered from some significant methodological limitations which restricts efficacy in terms of policy implementation. First, much of the evidence is cross-sectional which limits the degree to which the observed associations can be considered causal, a key consideration for policy-makers. For example, it may be the case that tobacco retailers preferentially locate in areas with greater demand resulting in a causal pathway that is in the opposite direction to that hypothesised. Furthermore, despite many studies including adjustment for a wide range of individual-level factors, it is unlikely that they remove all confounding associated with differences between individuals that may be related to both smoking propensity and the likelihood of living in areas with greateravailability of tobacco.^13^ Second, few studies have been able to incorporate analyses of *both* the tobacco supply environment and measures of neighbourhood smoking social norms, and it is therefore difficult to separate the influence of retail availability from local cultural and behavioural factors. This is despite recognition of the importance of the social context in determining smoking related behaviours.^14^ Areas with prosmoking attitudes are likely to see increase in supply (new retailers) through increased demand for tobacco products at the same time as increasing the risk of individual smoking. Finally, despite the recognition that pregnant women are a key target group for improving health and well-being and reducing inequalities among women and children, there has been surprisingly little previous work in the literature on the environmental influences on smoking behaviours among this key group.

Administrative maternity records in Scotland, which cover the entire population of pregnancies, include an indication of maternal smoking during pregnancy and therefore provide an opportunity to address these limitations. First, we can use the full population coverage of these data to estimate neighbourhood influences on smoking behaviour allowing us to examine whether tobacco retailing is independent of neighbourhood level patterns of smoking behaviour. Second, through an analytical approach that is closer to an experimental design, we can address issues of confounding, reverse causation and the non-exchangeabillity of exposure groups which are typical of cross-sectional analyses. We do this by comparing smoking behaviour across multiple pregnancies to the same mother against changes in exposure to tobacco retailer density from residential moves so that individuals act as their own exposure controls. Within this quasi-experimental framework, we aim to: (1) establish whether changes in exposure to local tobacco retail environments influence changes to smoking behaviour during pregnancy and (2) determine if these associations are attenuated after controlling for a measure of neighbourhood maternal smoking prevalence.

## Methods

### Study design

In addition to standard maternal clinical and demographic information, administrative maternity records in Scotland record smoking behaviour during pregnancy together with residential location at the time of delivery. Because the records are collected for administrative purposes, these data effectively provide individual longitudinal information on smoking behaviour during pregnancy for the entire population of mothers experiencing at least two pregnancies. Potentially confounding mother-level associations can be removed by examining changes in smoking behaviour within mothers (between pregnancies) to changes in exposure (from residential moves occurring between pregnancies). In contrast to a cross sectional analysis, this approach makes comparisons between the same mother at different points in time rather than between different mothers at the same point in time. This approach is powerful because any confounding effects that arise from individual differences that do not change over time are removed by design thereby removing a considerable source of confounder bias from the analysis.^15^ A key element of the analysis is that only a subset of pregnancies (hereafter referred to as the analysis sample) are used to estimate the model. This is because individual mothers who do not experience changes in outcome (smoking status) contain no variation and therefore contribute no information to help the estimation and are therefore discarded. Our analysis sample therefore contains only those individuals whose outcome and exposure change between pregnancies (ie, those mothers who have had, in the data period, at least two pregnancies and have changed smoking status at least once between those pregnancies). The main drawback of this approach is reduced statistical efficiency and in order to power a fixed-effects model adequately, large samples of repeated measures are required. The use of population administrative data can address this issue permitting a within-mother study design. Moreover, this data also allow the influence of neighbourhood maternal smoking prevalence to be examined by calculating prevalence of smoking during pregnancy in small areas across Scotland.

### Maternity records and smoking outcome data

We obtained maternity hospital records for all births in Scottish National Health Service (NHS) hospitals in the period 2000–2015 (n=8 41 252) from the Scottish Morbidity Record (SMR02). The SMR has approximately 98% population capture with the missing 2% made up of births outside of NHS hospitals including home births. Of these, we removed 17 655 records to exclude births to mothers under the age of 18, 67 957 records that were missing smoking information, 1995 records that were missing information for tobacco retailer density and an additional 450 records that were missing information for other covariates. From the remaining 753 195 records, 697 961 (93%) were not considered in the analysis because they were to mothers who either had only one pregnancy in the period or who did not change smoking behaviour between multiple pregnancies leaving 55 234 (7%) pregnancies in the analysis sample.

Self-reported smoking behaviour is collected during the antenatal booking appointment (which takes place at the end of the first trimester at around 8–12 weeks) and is recorded by the midwife as being either a current, former or never smoker. An ‘unknown’ category was used for those women whose status was not recorded. The question and method of collection in the SMR records has remained consistent over time. We derived a binary outcome variable from this measure with current smokers coded 1 and former or never smokers coded 0. Those with unknown smoking status were excluded from the analysis.

### Tobacco outlets and neighbourhood maternal pregnancy smoking prevalence

Assessment of the tobacco retail environment is based on the same method and data as adopted in earlier work in Scotland.^16^ In short, addresses of all premises registered on the Scottish Tobacco Retailers Register as at 30 September 2012 are obtained and georeferenced. The whole of Scotland is then transformed into a continuous surface of grid cells (100×100 m in size). Using kernel density estimation, each cell is assigned a value designed to capture the distance-weighted density of retailers within an 800 m buffer. Buffers of 400 m and 1000 m were considered in sensitivity analysis. The density measures (measured in units of retailers per km^2^) were linked to the individual-level pregnancy records by using each women’s postcode to identify the grid cell in which they were located at each pregnancy. Further details about the method of calculating retail density can be found in Shortt *et al*.^16^ We categorise the density variable into five groups with group 1 containing areas with zero retailer density and subsequent groups defined with breaks at the following values: >0 to ≤4.2, >4.2 to ≤10.5, >10.5 to ≤18.9 and >18.9.

The full population of maternity records provide sufficient numbers in each geographical area to calculate an annual geographical smoking prevalence measure. Neighbourhood prevalence has been shown to be associated with not just individual smoking behaviours but also positive perceptions of smoking.^17^ In the absence of information about neighbourhood social and cultural norms of smoking among pregnant women, it is plausible that this measure may capture some of these neighbourhood smoking effects. It also captures the degree of ‘visibility’ of smoking among other pregnant women in the neighbourhood including during interactions with antenatal and other healthcare services. Given that the catchment area of such services is likely to be larger than the size of a data zone (which are designed to contain between 500 and 1000 households and vary in size depending on population density), we choose to use a larger geography (intermediate data zones which contain on average 4000 households) for aggregation to capture this effect. Intermediate data zones also have the advantage of being large enough to contain sufficient cases per year to calculate a stable rate. This neighbourhood maternal smoking prevalence was categorised into groups with group one containing areas with zero neighbourhood prevalence and subsequent groups defined with breaks at the following values: >0% to ≤10%, >10% to ≤20%, >20% to ≤30% and >30%.

### Statistical methods

We estimated conditional logistic regression models with individual mother-level fixed effects to examine the risks of smoking during pregnancy adjusting for potentially confounding factors. We examined retailer density as both a categorical (for consistency with previous studies) and continuous variable in order to test for problems associated with the categorisation of continuous variables.^18^ In the continuous models, to capture potential non-linear relationships, we allow the functional form to vary using polynomial curve functions with fractional powers. These are a class of curves that are more flexible than traditional polynomial curves but less prone to overfitting than local smoothing techniques. The best fitting curve function was determined based on deviance reduction and plotted with a 95% CI.

### Covariates

The fixed-effects approach removes the need for adjustment of time-invariant individual level characteristics but the possibility of residual confounding by factors that vary over time remains. Confounding variables and appropriate ’blocking' variables were identified using a directed acyclic graph (DAG) (online supplementary figure A1), and based on this conceptual model we adjust for the following potentially time varying factors; year of delivery, area income deprivation (using the income domain of the Scottish Index of Multiple Deprivation; SIMD), sixfold urban and rural residential location, mothers age and neighbourhood maternal smoking prevalence. The SIMD is an aggregate area based measure that uses administrative data to estimate levels of deprivation within data zones across Scotland. The income domain is one component of the index and measures the proportion of all households requiring state income support in each data zone. These areas are ranked and then grouped into quintiles. Urban and rural residential location is determined using the Scottish Government classification which is based on two criteria, population size and drive-time accessibility, which are combined to group data zones across the whole of Scotland into six urban and rural categories (More details about the precise definitions are available at http://www.gov.scot/Topics/Statistics/About/Methodology/UrbanRuralClassification). Age was categorised with 5-year age bands apart from at the youngest ages^18 19^ which were separated in order to better capture different effects for younger mothers. With the exception of year of delivery (which is included in the model as a continuous trend), all covariates are included in the models as categorical variables with categories as defined in table 1.

10.1136/tobaccocontrol-2018-054422.supp1 Supplementary data

**Table 1:** Pregnancy-level descriptive statistics for both exposures (retailing density and neighbourhood maternal smoking prevalence) broken down by smoking status

	Smoking status		
	No (row %)	Yes (row %)	Total (Col %)
Tobacco retailer density (outlets per km^2^)			
Group 0 (zero outlets)	1558 (53.67)	1345 (46.33)	2903 (5.26)
Group 1 (>0 to ≤4.2)	12 246 (50.00)	12 244 (50.00)	24 490 (44.34)
Group 2 (>4.2 to ≤10.5)	10 070 (48.60)	10 650 (51.40)	20 720 (37.51)
Group 3 (>10.5 to ≤18.9)	2053 (46.88)	2326 (53.12)	4379 (7.93)
Group 4 (>18.9)	1258 (45.88)	1484 (54.12)	2742 (4.96)
Neighbourhood maternal smoking prevalence			
Group 0 (zero prevalence)	374 (59.27)	257 (40.73)	631 (1.14)
Group 1 (>0% to ≤10%)	4030 (56.29)	3129 (43.71)	7159 (12.96)
Group 2 (>10% to ≤20%)	7151 (51.10)	6844 (48.90)	13 995 (25.34)
Group 3 (>20% to ≤30%)	7771 (49.82)	7826 (50.18)	15 597 (28.24)
Group 4 (>30%)	7859 (44.02)	9993 (55.98)	17 852 (32.32)
Scottish Index of Multiple Deprivation			
Quintile 1 (most deprived)	6244 (46.66)	7137 (53.34)	13 381 (24.23)
Quintile 2	6834 (48.20)	7373 (51.90)	14 207 (25.72)
Quintile 3	6096 (50.16)	6058 (49.84)	12 154 (22.00)
Quintile 4	4677 (50.49)	4586 (49.51)	9263 (16.77)
Quintile 5 (least deprived)	3334 (53.52)	2895 (46.48)	6229 (11.28)
Mothers age			
18–19	2058 (44.11)	2608 (55.89)	4666 (8.45)
20–24	7026 (43.73)	9039 (56.27)	16 065 (29.09)
25–29	8063 (49.15)	8341 (50.85)	16 404 (29.70)
30–34	6389 (53.16)	5630 (46.84)	12 019 (21.76)
35–39	3106 (59.31)	2131 (40.69)	5237 (9.48)
40–44	525 (63.95)	296 (36.05)	821 (1.49)
45+	18 (81.82)	4 (18.18)	22 (0.04)
Urban and rural			
Large urban	8411 (48.62)	8887 (51.38)	17 298 (31.32)
Other urban	11 108 (48.94)	11 590 (51.06)	22 698 (41.09)
Accessible small town	2684 (49.81)	2705 (50.19)	5389 (9.76)
Remote small town	1108 (48.49)	1177 (51.51)	2285 (4.14)
Accessible rural	2709 (51.41)	2560 (48.59)	5269 (9.54)
Remote rural	1165 (50.76)	1130 (49.24)	2295 (4.16)
Total	27 185 (49.22)	28 049 (50.78)	55 234 (100)

### Results

A total of 55 234 pregnancies were to women who had at least two pregnancies in the period 2000–2015 and were discordant in smoking status in at least one of these pregnancies. These pregnancies occurred to 22 927 individual mothers of which 69% had two pregnancies in the period, 24% had three and 8% had four or more. Sixty-eight per cent of these mothers moved at least once across these multiple pregnancies. Table 1 presents pregnancy level descriptive statistics for exposures and covariates broken down by smoking status and shows smoking rates decreasing with age and increasing with area based deprivation. Table 2 presents descriptive statistics for the continuous exposure measures.

**Table 2:** Descriptive statistics for continuous exposure measures

	Smoking pregnancies		Non-smoking pregnancies	
	Mean	SD	Mean	SD
Tobacco retailer density (outlets per km^2^)	6.25	7.37	5.91	7.26
Neighbourhood prevalence (proportion of intermediate data zone)	0.38	0.19	0.22	0.18

**Figure 1:**
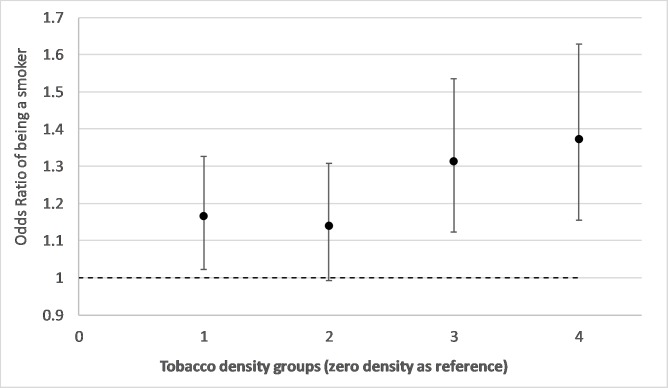
Adjusted ORs and 95% CIs from conditional logistic regression models of smoking during pregnancy associated with neighbourhood tobacco retailer density groups(group 4 represents an area of the highest density) adjusting for mothers age, urban and rural residence, birth year trend and Scottish Index of Multiple Deprivation income deprivation quintiles.

**Figure 2:**
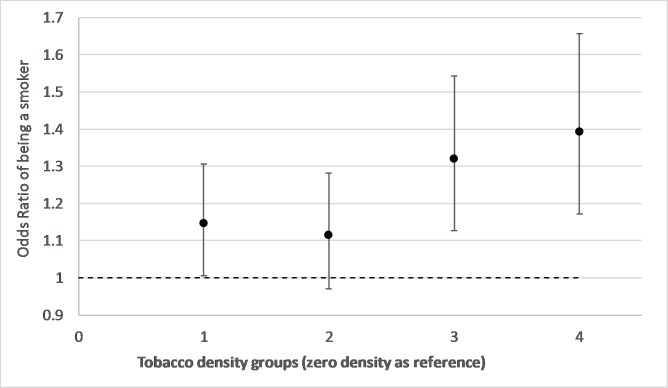
Adjusted ORs and 95% CIs from a conditional logistic regression model of smoking during pregnancy associated with neighbourhood tobacco retailer density groups (group 4 represents an area of the highest density). The model adjusts for mothers age, urban and rural residence, birth year trend, Scottish Index of Multiple Deprivation income deprivation quintiles and neighbourhood maternal smoking prevalence groups.

**Figure 3:**
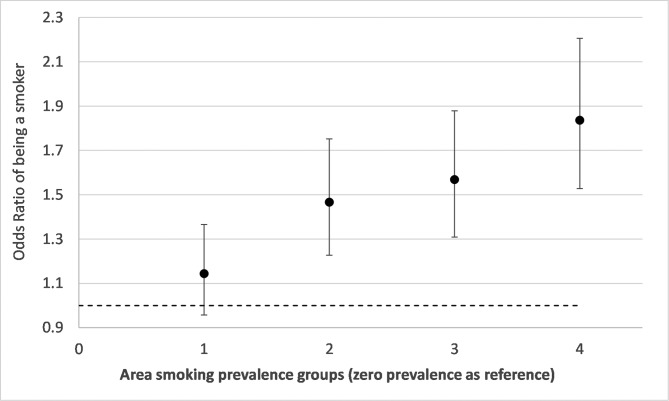
Adjusted ORs and 95% CIs from a conditional logistic regression model of smoking during pregnancy associated with neighbourhood maternal smoking prevalence groups (group 4 represents an area of the highest prevalence). The model adjusts for mothers age, urban and rural residence, birth year trend, Scottish Index of Multiple Deprivation income deprivation quintiles and neighbourhood tobacco retailer density groups.

Figure 1 shows ORs from a model of the relationship between tobacco retailer density groups and risks of smoking during pregnancy with adjustment for year of delivery (trend), mothers age, SIMD area income deprivation and urban/rural residence. It shows an increase in the risk of smoking during pregnancy for groups 1, 3 and 4 relative to the zero density category. Group 4 shows a 37% (OR 1.37, CI 1.15 to 1.63, p=0.00) increased risk of being a smoker compared with individuals living in areas with zero density. Figures 2 and 3 show results from the same model but with additional adjustment for neighbourhood maternal smoking prevalence. Figure 2 shows increases in the risk of smoking for retailer density groups 1, 3 and 4. Compared with figure 1, there is a slight strengthening of the observed associations with an excess risk of 39% (OR 1.39 CI 1.17 to 1.66, p=0.00) for group 4. Figure 3 shows the effect of neighbourhood maternal smoking prevalence from the same model. It shows statistically significant increases in risks of smoking in groups 2, 3 and 4 relative to group 0 with group 4 showing an excess of risk of 84% (OR 1.84 CI 1.53 to 2.21, p=0.00). In both models, age, reduced area income deprivation and living in less remote areas all reduced the risks of being a smoker (online supplementary table A3).

**Figure 4:**
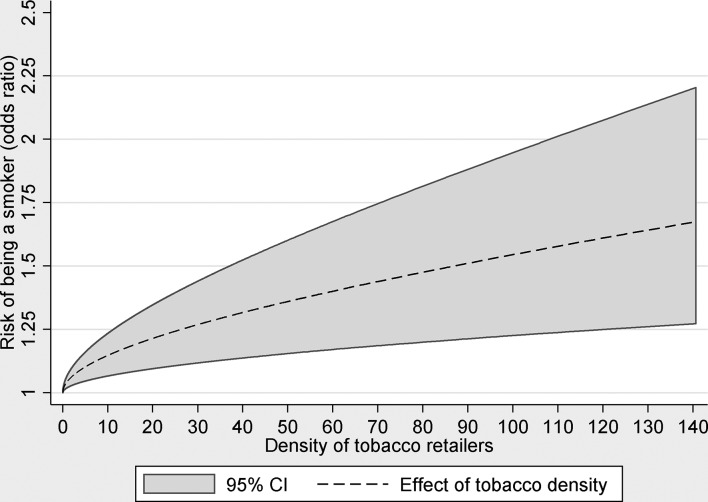
Adjusted OR and 95% CIs from conditional logistic regression models of smoking during pregnancy associated with neighbourhood tobacco retailer density (outlets per km^2^) modelled as a continuous fractional polynomial function adjusting for mothers age, Scottish Index of Multiple Deprivation income deprivation quintiles and neighbourhood smoking prevalence. The polynomial function shows change in OR of smoking relative to zero retailer density across the range of density values that are present in the data.

Figure 4 presents results from a model with tobacco density as a continuous variable allowing the shape of the relationship to be a non-linear function. With adjustment for year of delivery (trend), mothers age, SIMD area income deprivation, urban/rural residence and neighbourhood maternal smoking prevalence groups, the graph shows a positive relationship between tobacco retail density and odds of smoking with ORs increasing to 1.67 (95% CI 1.27 to 2.20) in the highest density areas relative to areas with zero density. The relationship is slightly non-linear with steeper increases in risk at lower densities and smaller increases in risk in higher density areas.

## Discussion

This study has found strong and significant positive associations between residential density of tobacco retailers and the risk of maternal smoking during pregnancy. We estimate that a mother who is pregnant living in a neighbourhood with the highest density of tobacco retailers is around 67% more likely to be a smoker than a pregnancy to the same mother occurring in a neighbourhood with the lowest retailer density. To date, research examining the influence of the local density of tobacco retailing on tobacco consumption and related health outcomes has largely relied on evidence from cross-sectional studies and has not incorporated a measure of neighbourhood smoking prevalence. The findings for smoking during pregnancy are novel but are consistent with previous cross-sectional studies from other population groups.^3 4 19 20^ We argue that the findings in this study constitute the strongest evidence to date because they are based on longitudinal changes in smoking within individuals and are independent of a measure of neighbourhood maternal smoking prevalence. Reducing the local density of tobacco retailers has been identified as a ‘new frontier’ of tobacco control,^2^ and the current study significantly extends the evidence base supporting reductions in tobacco availability and supply as a means of tackling tobacco use.

The findings are especially timely given the very recent publication by the UK government of the new tobacco control strategy for England and Wales which makes almost no mention of retail availability as a point of intervention.^21^ In the context of Scotland, the findings provide important evidence that intervening in tobacco supply may assist in efforts towards the tobacco ‘endgame’ and the target of achieving smoking prevalence below 5% by 2034. The current Tobacco Control Strategy for Scotland (2013) has acknowledged the need to ‘create an environment where young people choose not to smoke’.^22^ Scotland, demonstrating political leadership in public health, has led on policy to become tobacco free, including the introduction of smoke-free places. Smoking rates in Scotland have fallen from 31% in 1991 to 23% in 2012.^23^ This rate has however remained static since 2013,^24^ supporting the argument that ‘tobacco endgames’ must now go beyond ‘business as usual’.^25^ Such a move requires innovative policy solutions that adjust the tobacco retailing environment. A collection of researchers, advocates, policy-makers and practitioners based in Scotland have proposed four broad policy proposals, one of which was to apply measures to reduce the local provision of tobacco retailing.^26^ Similar proposals have been developed in New Zealand.^27^ The results of this research add further weight to this call. A refresh of the Scottish Tobacco Control Strategy will be published in the summer of 2018. We propose that such a strategy should consider practical means to reduce the ubiquitous availability of tobacco in our communities.

Such a move would also support the Scottish Governments commitment to tackling health inequalities. Given that the highest levels of tobacco retailer density are more likely to be in more deprived areas, and that those living in areas of highest density are less likely to successfully quit,^28^ such an intervention is also likely to contribute to a reduction in health inequalities. Providing evidence to underpin the development of new policy responses that reduce smoking prevalence among pregnant women is especially important, particularly as many previous interventions have shown mixed success.^29 30^ Furthermore, the findings come at a key time in the UK where smoking during pregnancy has been identified as an important factor in fetal and infant morbidity^31^ and a priority area for action in improving and reducing inequalities in child health.^32^

The degree to which our results are generalisable to the whole population of pregnant women should be considered. Although the analytical design of the study is closer to the experimental ideal than a cross-sectional analysis, our analytical sample is restricted to the group of pregnant women who have had at least two pregnancies and who have changed smoking status at least once between these pregnancies. These individuals may not be comparable with those who are consistent in their smoking behaviour over time who may be either long term smokers or those who never or only irregularly smoke. In this latter group, the ‘opportunity effect’ and visual cues of tobacco availability is unlikely to be as important when compared with our analysis sample, a greater proportion of which will be individuals who are or have been trying to quit smoking.

The use of self-reported smoking information remains a limitation of the data used in this study. Evidence suggests that around 2400 pregnant smokers a year in Scotland are undetected when prevalence is estimated based on self-reports, a pattern which is strongly associated with area deprivation.^33^ However, for this to bias the results of this study, the risk of under-reporting would have to be associated with outlet density independently of area deprivation. After accounting for deprivation, the distribution of mothers under-reporting smoking is likely to be random with respect to outlet density. Furthermore, it is likely that under-reporting of smoking is likely to be higher in areas with lower overall prevalence. We were also limited to measuring retailer density at one point in time (2012). However, evidence suggests that there has been little change in retailer densities over time in Scotland in the period 2010–2017, and little in the way of public health interventions to restrict tobacco retailers before this period. Furthermore, any unmeasured temporal variation that is present is likely to be randomly distributed with respect to this study and is therefore unlikely to significantly affect the results.

Despite the strengths of the analytical design of the study, there remain other limitations with our analysis. First, we assume that postcode at delivery was the same as postcode throughout the 9-month period of pregnancy and, second, the analysis was unable to incorporate analyses of consumption patterns or purchasing behaviour and how these may be influenced by the retailer environment.

Although the analytical design of the study fully controls for confounding at the individual level for factors that do not vary over time, there remains the potential for confounding from factors that change over time that may also be related to both propensity to smoke as well as residential movement to areas with greater densities of tobacco retailers. For a variable to be a confounder, it must be time varying and it must be associated with changes in tobacco density exposure and changes in smoking outcome. For example, stressful life events and changes in life circumstances such as unemployment or relationship breakdown may be associated with both smoking behaviour and movement to an area with higher tobacco outlet densities. However, if our conceptual model, as set out in the DAG (online supplementary figure A1) is correct, then adjustment for residential moves to a more deprived area (measured by SIMD area income deprivation) removes the confounding pathways associated with these stressful life events. Although changes in these unmeasured variables contain a direct pathway to smoking, they are not directly connected to tobacco outlet density apart from via a pathway through changes in area income deprivation (through residential relocation). Adjusting for changes in area income deprivation therefore means the observed association between outlet density and smoking is independent of changes in the unobserved variables. While other sources of time varying confounding may exist, they are unlikely to account for the associations we have observed.

We also conceptualise neighbourhood smoking prevalence as a confounder. Areas with higher smoking prevalence are likely to be identified by retailers as areas of higher demand increasing the likelihood of locating there. This creates a confounding pathway between outlet density and smoking. However, it is equally likely that area smoking prevalence may in part be determined by outlet density and therefore may constitute a mediator rather than a confounder of the outlet density association. Thus, adjustment for area smoking prevalence may constitute overadjustment of the outlet density association. Although our results show that both exposures play a role in mediating smoking behaviour independently of each other (with area prevalence associations being the stronger of the two), policy interventions that tackle the supply of tobacco may in time reduce area prevalence by reducing the effects associated with retailing density and supply.

To the best of our knowledge, this is the first study to examine the influence of neighbourhood tobacco availability on smoking behaviour during pregnancy and one of the few studies of tobacco availability to incorporate a longitudinal analysis with a large dataset. Our results show the significant role of the tobacco retailer environment in influencing smoking during pregnancy. The findings are the strongest indication yet of the need for policy to tackle the supply side of tobacco usage as a means of meeting the commitments laid out in national tobacco control strategies and of reducing smoking prevalence.

**What this paper adds**

**What is already known on this subject**

- The density of tobacco retailers in residential, school and other settings has been shown to be associated with the risk of being a smoker.
- These associations are consistent across different sociodemographic groups.

**What important gaps in knowledge exist on this topic**

- A causal interpretation is limited because previous evidence is almost entirely cross-sectional and may suffer from issues of reverse causality and residual confounding.
- Among existing studies of tobacco retailer density and smoking behaviour, none have examined the relationship with smoking during *pregnancy* and none have controlled for a measure of neighbourhood maternal smoking prevalence.

**What this paper adds**

- With full population data, we examine *within-individual* changes in pregnancy smoking in relation to changes in exposure to neighbourhood tobacco availability controlling for neighbourhood maternal smoking prevalence.
- Risks of pregnancy smoking increased significantly when the pregnancy occurred in areas with the highest availability of tobacco compared with a pregnancy to the same individual in an area with zero tobacco availability.
- The findings are unique with respect to smoking during pregnancy and the availability of tobacco and affirm and extend previous cross-sectional evidence from the general population and other demographic groups.
- National tobacco strategies should incorporate policies restricting tobacco supply as a means of tackling population tobacco use.
