# Supplementary material for: Neighbourhood tobacco supply and individual maternal smoking during pregnancy: a fixed-effects longitudinal analysis using routine data
Source: Tob Control. 2018 Nov 2;29(1):7–14. doi: 10.1136/tobaccocontrol-2018-054422 (PMC6952837; doi:10.1136/tobaccocontrol-2018-054422)
Supplement: Supplementary data [file tobaccocontrol-2018-054422supp001.pdf]

Appendix

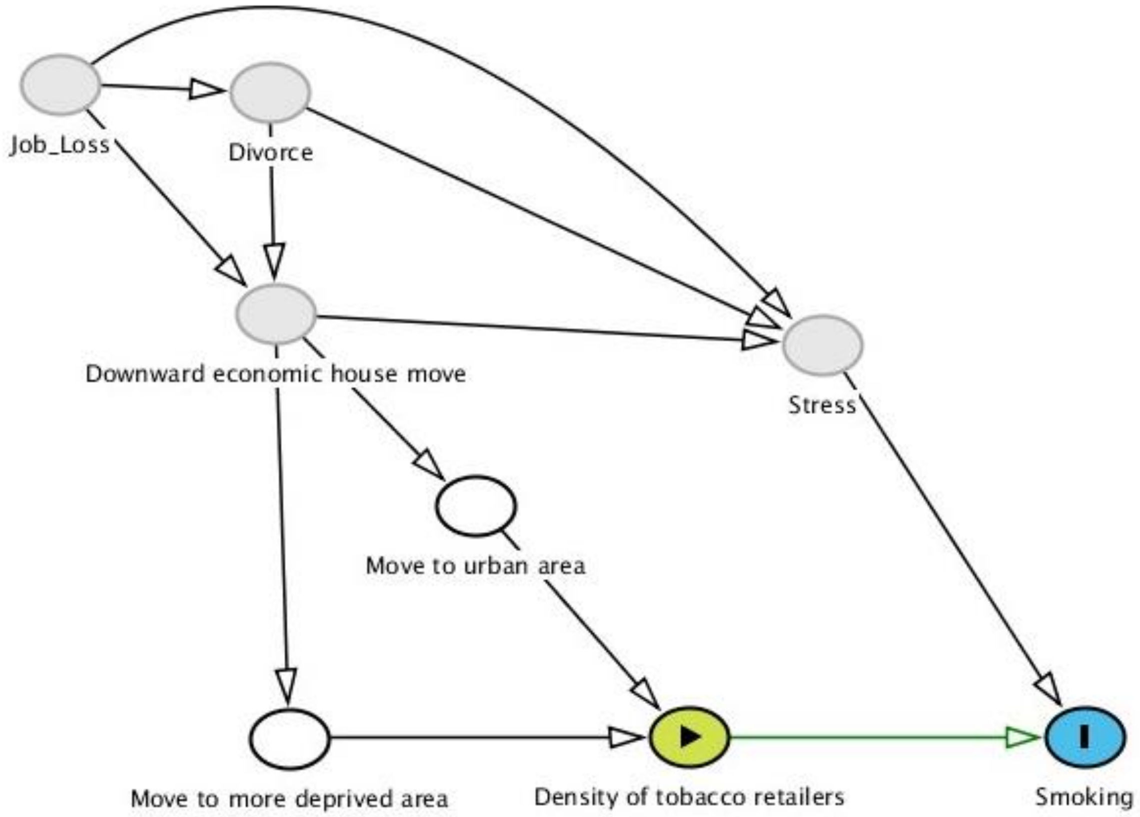

Figure A1: Our proposed DAG representation of the underlying causal structure. We argue, based on this particular conceptual structure, that adjustment for the variables labelled with white circles removes confounding due to unmeasured variables (represented in grey circles) leaving an unconfounded pathway (green arrow path) between the green circle (change in exposure to tobacco outlet density) and the outcome in the blue circle (change in smoking)

Table A1: Pregnancy-level descriptive statistics for both exposure (retailing density) and covariates for the full population of pregnancies in Scotland.

	**Full Population**		
	**Smoking Status**		
	**No (row %)**	**Yes (row %)**	**Total (Col %)**
**Tobacco Retailer Density (outlets per km^2)**			
Group 0 (Zero Outlets)	51,869 (88.60)	6,672 (11.40)	58,541 (7.77)
Group 1 (>0 - <=4.2)	274,665 (79.42)	71,169 (20.58)	345,834 (45.92)
Group 2 (>4.2 - <=10.5)	174,514 (72.90)	64,877 (27.10)	239,391 (31.78)
Group 3 (>10.5 - <=18.9)	46,725 (77.08)	13,893 (12.92)	60,618 (8.05)
Group 4 (>18.9)	39,948 (81.84)	8,863 (18.16)	48,811 (6.48)
**Neighbourhood Maternal Smoking Prevalence**			
Group 0 (Zero prevalence)	17,950 (93.78)	1,190 (6.22)	19,140 (2.54)
Group 1 (>0% - <=10%)	135,701 (90.04)	15,015 (9.96)	150,716 (20.01)
Group 2 (>10%- <=20%)	174,362 (82.56)	36,831 (17.44)	211,193 (28.04)
Group 3 (>20% - <=30%)	141,613 (75.46)	46,060 (24.54)	187,673 (24.92)
Group 4 (>30%)	118,095 (64.02)	66,378 (35.98)	184,473 (24.49)
**SIMD**			
Quintile 1 (Most deprived)	80,272 (61.62)	49,993 (38.38)	130,265 (17.29)
Quintile 2	108,027 (70.09)	46,096 (29.91)	154,123 (20.46)
Quintile 3	121,152 (78.06)	34,053 (21.94)	155,205 (20.61)
Quintile 4	133,934 (85.56)	22,602 (14.44)	156,536 (20.78)
Quintile 5 (Least deprived)	144,336 (91.90)	12,730 (8.10)	157,066 (20.85)
**Mothers age**			
18-19	20,297 (56.19)	15,828 (43.81)	36,125 (4.80)
20-24	90,363 (64.84)	48,994 (35.16)	139,357 (18.50)
25-29	159,536 (78.03)	44,921 (21.97)	204,457 (27.15)
30-34	191,376 (84.79)	34,325 (15.21)	225,701 (29.97)
35-39	104,749 (85.57)	17,664 (14.43)	122,413 (16.25)
40-44	20,496	3,631	24,127

	(84.95)	(15.05)	(3.20)
45+	904<br>(89.06)	111<br>(10.94)	1,015<br>(0.13)
**Urban and Rural**			
Large Urban	209,001<br>(79.31)	54,518<br>(20.69)	263,519<br>(34.99)
Other urban	213,889<br>(75.62)	68,970<br>(24.38)	282,859<br>(37.55)
Accessible small town	53,772<br>(77.70)	15,436<br>(22.30)	69,208<br>(9.19)
Remote small town	17,798<br>(73.02)	6,577<br>(26.98)	24,375<br>(3.24)
Accessible rural	65,591<br>(82.94)	13,489<br>(17.06)	79,080<br>(10.50)
Remote rural	27,670<br>(81.02)	6,484<br>(18.98)	34,154<br>(4.53)
**Total**	587,721<br>(78.03)	165,474<br>(21.97)	753,195<br>(100)

Table A2: Pregnancy level descriptive statistics for continuous exposures and covariates for the full population of pregnancies in Scotland.

	**Smoking pregnancies**		**Non-smoking pregnancies**	
	**Mean**	**SD**	**Mean**	**SD**
**Tobacco Retailer Density (outlets per km^2)**	6.34	7.29	6.19	8.75
**Neighbourhood prevalence (proportion of intermediate datazone)**	0.40	0.19	0.17	0.17

Table A3: Full model outputs for conditional logistic regression models of smoking during pregnancy associated with neighbourhood tobacco retailer density quintiles (quintile 4 represents an area of the highest density) adjusting for mothers age, urban and rural residence, birth year trend and SIMD income deprivation quintiles (model 1) and additionally Neighbourhood Maternal Smoking Prevalence (model 2).

	Model 1		Model 2	
	Odds Ratio	95% CI	Odds Ratio	95% CI
**Tobacco Retailer Density (outlets per km^2)**				
Group 1 (>0 - <=4.2)	1.16	1.02,1.33	1.15**	1.01,1.31
Group 2 (>4.2 - <=10.5)	1.14	0.99,1.31	1.12	0.97,1.28
Group 3 (>10.5 - <=18.9)	1.31	1.12,1.54	1.32***	1.13,1.54
Group 4 (>18.9, Highest density)	1.37	1.15,1.63	1.39***	1.17,1.66
**Neighbourhood Maternal Smoking Prevalence**				
Group 1 (>0% - <=10%)			1.14	0.96,1.37
Group 2 (>10%- <=20%)			1.47***	1.23,1.75
Group 3 (>20% - <=30%)			1.57***	1.31,1.88
Group 4 (>30%, Highest prevalence)			1.84***	1.53,2.21
**Year of Delivery**	0.94	0.92,0.95	0.94***	0.93,0.95
**Mothers age**				
20-24	1.20***	1.11,1.3	1.19***	1.11,1.29
25-29	1.18**	1.05,1.33	1.17**	1.04,1.32
30-34	1.08	0.91,1.28	1.07	0.90,1.27
35-39	0.85	0.68,1.07	0.85	0.67,1.06
40-44	0.67**	0.49,0.91	0.66**	0.48,0.91
**SIMD Area Income Deprivation**				
Quintile 2	0.95	0.88,1.01	0.99	0.92,1.06
Quintile 3	0.88***	0.82,0.96	0.97	0.89,1.05
Quintile 4	0.94	0.86,1.02	1.07	0.98,1.18
Quintile 5 (Least deprived)	0.83***	0.75,0.92	1.02	0.91,1.14
**Urban and Rural**				
Other urban	1.00	0.9,1.12	0.97	0.87,1.09
Accessible small town	0.90	0.78,1.03	0.87*	0.76,1.00
Remote small town	1.22*	0.98,1.51	1.15	0.93,1.43
Accessible rural	0.96	0.84,1.11	0.96	0.83,1.10
Remote rural	1.24**	1.01,1.54	1.24*	1.00,1.53

*** $p < 0.01$ ** $p < 0.05$ * $p < 0.1$
